# Lateral Root Development in Potato Is Mediated by Stu-mi164 Regulation of NAC Transcription Factor

**DOI:** 10.3389/fpls.2018.00383

**Published:** 2018-03-29

**Authors:** Li Zhang, Lei Yao, Ning Zhang, Jiangwei Yang, Xi Zhu, Xun Tang, Alejandro Calderón-Urrea, Huaijun Si

**Affiliations:** ^1^Gansu Provincial Key Laboratory of Aridland Crop Science, Gansu Key Laboratory of Crop Genetic and Germplasm Enhancement, Gansu Agricultural University, Lanzhou, China; ^2^College of Life Science and Technology, Gansu Agricultural University, Lanzhou, China; ^3^College of Plant Protection, Gansu Agricultural University, Lanzhou, China; ^4^Department of Biology, California State University, Fresno, CA, United States

**Keywords:** miRNA, NAC, osmotic stress, lateral root, potato

## Abstract

The NAC designation is derived from petunia (*Petunia hybrida*) gene *NO APICAL MERISTEM* (*NAM*) and *Arabidopsis* genes *ATAF1/ATAF2* and *CUP-SHAPED COTYLEDON2* (*CUC2*), which belongs to the family of plant-specific transcription factors (TFs), and plays important role in plant development processes, such as response to biotic and abiotic stress, and hormone signaling. MicroRNAs (miRNAs) are a class of small, non-coding endogenous RNAs which play versatile and significant role in plant stress response and development via negatively affecting gene expression at a post-transcriptional level. Here, we showed that Stu-mi164 had a complementary sequence in the CDS sequence of potato NAC TFs, and that NAC expression exhibited significant differences under osmotic stress. We measured expression levels of the Stu-mi164 target gene *StNAC262* between control and PEG-treated plants using real-time PCR, and the results demonstrated that they had inverse relationship. We suggested that Stu-miR164 might drive overexpression of *NAC* gene under osmotic stress in potato. To confirm the regulation of NAC TFs by Stu-mi164, we developed transgenic plants, using *Agrobacterium tumefaciens*–mediated transformation, of the potato cultivars “Gannongshu 2” and “Kexin 3” overexpressing the Stu-mi164 or the TF *StNAC262*. Real-time PCR analysis of transgenic potato plants under osmotic (PEG) stress, showed that potato plants overexpressing Stu-mi164 had reduced expression of *StNAC262* and their osmotic resistance decreased. Furthermore, these plants had low number of lateral roots although the same length as the control. Our findings support the regulatory role of Stu-miRNAs in controlling plant response to osmotic stress via *StNAC262*.

## Introduction

Drought stress is one of the main factors severely affecting plant physiology and biochemistry, ultimately limiting plant growth and development and causing decrease in crop production efficiency (Thao and Tran, 2012). Plants have developed sophisticated mechanisms to deal with diverse unfavorable environmental factors. A number of physiological and metabolic processes of plant were triggered to promote plant survival under water deficit (Shinozaki and Yamaguchi, 2007). Numerous genes of plant, including transcription factor (TF) genes, have altered their expression for stress adaptation (Yang et al., 2010). TFs play vital role in stress responses of plant by binding to the *cis*-acting element located in promoter region of downstream target genes to regulate various signaling pathways, thereby activating them, and by interacting with other proteins (Yamaguchi-Shinozaki and Shinozaki, 2006).

About 20 years ago, the specific NAC TF family of plant was first described in *Petunia* (Souer et al., 1996). Many studies have found that most of the *NAC TFs* genes are influenced by a variety of biological and abiotic stress which shows that they are plays important roles in stress response and signal transduction (Tran et al., 2004; Liu et al., 2011). We deleted the reference Chatterjee et al., 2011. The genomic sequencing results allowed identifying NAC family members in a number of sequenced species, for example, 163 genes in poplar (*Populus trichocarpa*) (Hu et al., 2010), 152 genes in tobacco (*Nicotiana tabacum*) (Rushton et al., 2008), 151 genes in rice (*Oryza sativa*) (Nuruzzaman et al., 2010), 117 genes in *Arabidopsis* and about 200 genes in soybean (*Glycine max*) (Mochida et al., 2009). The NAC TFs are multi-functional proteins which are involved in response to diverse cues, including biotic and abiotic stress responses (Olsen et al., 2005), lateral root formation and auxin signaling (Xie et al., 2000), secondary cell wall synthesis regulation, cell division (Zhong et al., 2007), embryo development (Duval et al., 2002), and flowering (Yoo et al., 2007). The NAC TFs have a conserved DNA-binding domain and a variable domain at the end N-terminal and C-terminal end, respectively, which are important for the transcriptional regulatory roles (Duval et al., 2002).

Xie et al. (2000) found that *NAC1* gene in *Arabidopsis thaliana* was induced by auxin and mediated auxin signaling in order to promote development of lateral root. This TF can activate two downstream auxin response genes *DBP* (DNA binding protein) and *AIR3* (auxin-induced in root cultures 3) to express. Excessive expression of *NAC1* can promote lateral root development, while antisense expression inhibits lateral root development. *DBP* encodes a DNA binding protein and *AIR3* encoding a class of hay bacillus protease that can weaken the connections between cells, indirectly promoting the development of lateral roots (Alliotte et al., 1989). In addition, *Arabidopsis thaliana AtNAC2* gene is specifically expressed in the root after induction by ethylene signaling and auxin signaling pathways, which can significantly increase the number of lateral root (He et al., 2005). Moreover, NAC TF was induced by a variety of biotic stress, involved in plant stress response. Hu et al. (2006) cloned a rice drought resistance and salt tolerance gene *SNAC1*, which is a type of NAC TF expressed primarily in stomatal guard cells; it was proposed drought stress promote stomatal closure but does not affect the photosynthetic rate, thus greatly improving drought resistance. Using yeast single hybrid technology, Tran et al. (2004) isolated three different *NAC* genes from *Arabidopsis thaliana* (*ANAC019*, *ANAC055*, and *ANAC072*). Overexpression of three *NAC TFs* gene in *Arabidopsis thaliana* can enhance the drought-resistant ability of genetically modified plants. High expression levels of these genes in the transgenic plants induced by drought, high salt, and ABA, significantly enhanced the drought-resistant ability of the plants (Tran et al., 2004).

MicroRNAs (miRNAs) are small and endogenous RNAs, which play vital regulatory function in stress responses of plant through negatively affecting gene expression at the level of post-transcription (Voinnet, 2009), by degrading transcript of the target genes (Llave et al., 2002), and therefore play an important role in attenuating translation (Chen, 2004). miRNAs can bind to their target transcripts by complementary base pairing, and either direct the target cleavage or repress its translation, further leading to the decreased expression of the target gene; therefore, miRNAs can act at the levels of both transcription and post-transcription (Hikmet et al., 2015). There are few agronomic traits in crops are controlled by single gene or isolated biological pathway. Cellular responses to stresses usually involve complex networks of gene interaction which are regulated at multiple levels (Budak et al., 2015). In other plant species, conservation of miRNAs in sequence and structure provides strong characteristics for the prediction of new miRNAs (Kantar, 2010). To date, there are thousands of miRNAs and the majority of their target genes that have been identified. Many miRNA target transcripts have been associated with stress-responsive TF families including WRKY and NAC in *Triticeae* (Kantar et al., 2011; Deng et al., 2015). The target genes can encode important enzymes or TFs, which play important roles in plant development, including floral, stem, leaf and root development (Chen, 2004), signal transduction (Rhoades et al., 2002), auxin (Guo et al., 2005) and disease responses (Chapman et al., 2004), or various abiotic and biotic stresses (Jones-rhoades and Bartel, 2004). Most researches have been focused on model plants, such as rice (*Oryza sativa*) and *Arabidopsis thaliana* (Bartel and Bartel, 2003; Bonnet et al., 2004; Griffiths et al., 2006). Many conserved miRNAs of plant, such as miR156, miR159, or miR164, have been shown to target stress-related TFs including MYB and NAC family members (Burcu et al., 2016). In potato crop, few miRNAs have been found in drought stress or other abiotic stress responses (Hwang et al., 2011; Zhang et al., 2014). Moreover, there are no known miRNAs regulating NAC TFs under osmotic stress in potato.

We are interested in identifying novel miRNA genes that can regulate stress response and try to generate stress-resistant or tolerant potatoes by transgenic approach. In *Brassica* and rice seedlings, miR164 was shown to target a NAC TF, whose expression was negatively correlated with miR164 under stress conditions including drought, salinity, and high-temperature (Bhardwaj et al., 2014; Fang et al., 2014). In the present study, based on a miRNA bioinformatics analysis, we identified possible miRNAs that can regulate the *NAC* gene. We discovered that the potato Stu-mi164 had significant differences in expression levels under osmotic (PEG) stress. Target prediction also identified that it has a binding site on the CDS sequence of the *NAC* gene in potato. We further detected the expressional levels of the *NAC* gene response under osmotic (PEG) stress by qRT-PCR. We concluded that decreased expression of Stu-miR164 drives overexpression of the potato *NAC* gene and can help plant response to osmotic stress.

## Materials and Methods

### Plant Materials and Growth Conditions

The potato cultivars “Gannongshu 2” with more lateral roots and “Kexin 3” with fewer lateral roots were used in the experiment. The potato plantlets were propagated by sub-culturing using single-node cuttings on MS media containing 3% sucrose and 0.45% agar. Plantlets were grown in 150 mL flasks under 16/8 h light/dark cycle of white fluorescent lamp at (25 ± 2)°C. Microtubers were induced on MS media containing 8% sucrose and 0.45% agar under dark conditions at (25 ± 2)°C (Zhang et al., 2005).

### Bioinformatics and Software

The miRNA sequences of potato were obtained from the miRNA registry miRBase^1^ (Saini et al., 2008). The target genes of Stu-mi164 were predicted using online program psRNATarget^2^ (Dai and Zhao, 2011). WMD3^3^ (Web app for the automated design of artificial microRNAs) was use to design Stu-mi164 cloning primers. Sequence of potato and *Arabidopsis* NAC family TFs were obtained from plant TF database^4^ (Jin et al., 2014). The analysis of chromosome structure uses PGSC^5^. Alignment analysis and phylogenetic tree construction were achieved using CluxtalX 1.8 and MEGA 5.0 program. The data of qRT-PCR was analyzed by statistics software SPSS version 13.0.

### Subcellular Localization Assay

The coding sequence of *StNAC262* was amplified using gene-specific primers NAC262-F and NAC262-R and inserted into vector pEGFP at the *Bam*H I and *Xba* I sites, yielding plasmid pEGFP-NAC262. The plasmid pEGFP-NAC262 and pEGFP were transformed into *Agrobacterium tumefaciens* LBA4404 and the *agrobacteria* were infiltrated, separately, into tobacco (*Nicotiana benthamiana* L.) leaves using 1-mL needless syringes. After agro-infiltration, the plants were grown under room temperature (25°C) for 48 h in darkness. GFP fluorescence signals were excited at 488 nm and detected under a Leica SP8 confocal laser scanning microscope (Germany).

### Construction of the Plant Expression Vectors

The potato *StNAC262* gene sequence (GenBank accession number XM_006364522.1) was obtained from NCBI^6^ nucleic acid database. PCR primers were designed based on the *StNAC262* gene sequence using software Primer 5.0. The enzyme sites of *StNAC262* gene sequence were analyzed based the expression vectors pCPB121 and pCPB sequences to add the appropriate restriction sites *Xba* I, *Bam*H I, and protection bases at both ends of the primers. The PCR primers sequences as follows: StNAC262-F: TCTAGACCATCTCCTTCAAAGACCAC and StNAC262-R: GGATCCGCAGTGAGACAAATGGGAA (the underlined section showed enzyme sites). The Stu-mi164 primers was design on website named WMD3, and primer A (CTGCAAGGCGATTAAGTTGGGTAAC), B (GCGGATAACAATTTCACACAGG AAACAG), I (GATGGAGAAGCAGGGC ACATGCTTCTCTCTTTTGTATTCC), II (GAAGCATGTGCCCTGCTTCTCCATCAAAGAGAATCAATGA), III (GAAGA ATGTGCCCTGGTTCTCCTTCACAGGTCGTGATATG), and IV (GAAGGAGAA CCAGGGCACATTCTTCTACATATATATTCCT) (the underlined section showed the sequence of Stu-mi164) were used for overlapping PCR according to the method provided on the website to construct the Stu-mi164 cloning vector. The primers were synthesized by the Shanghai Biological Engineering Co., Ltd. The re-modified *StNAC262* gene and Stu-mi164 was driven by the constitutive CaMV 35S promoter. The engineered vectors, named pCPB-NAC262 and pCPB121-miR164, were, respectively, transformed into *Escherichia coli* DH5α, which was identified by double enzyme digested for further using. After identification, both of the two vectors were transformed into *Agrobacterium tumefaciens* LBA4404 by the freeze-thaw method (Wang et al., 2011).

### Potato Transformation and Identification of the Transgenic Plants

Potato transformation was performed using *Agrobacterium*-mediated method (Si et al., 2003). The microtubers of potato cultivars “Gannongshu 2” and “Kexin 3” were peeled and removed buds part, then cut into slices with thickness of 1–2 mm. The slices soaked into the *Agrobacterium* containing the vectors pCPB121-miR164 and pCPB-NAC262, after 7 min infection, the tissue slices were dried with filter paper placed into MS solid media at 28°C in the dark for 48 h. After co-cultivation the tissue slices were placed in differentiation media (MS + 1 mg/L IAA + 0.2 mg/L GA_3_ + 0.5 mg/L 6-BA + 2 mg/L ZT + 50 mg/L Kan + 500 mg/L carbenicillin), at 25°C for 2500 lx light to culture, and transferred every weeks to replace media. When the new buds were derived from the center of potato slice (Supplementary Figure S1a), they were transferred into rooting media containing 75 mg/L kanamycin and 200 mg/L carbenicillin for rooting screen about 7 days (Supplementary Figure S1b) when the buds were transferred to a selective rooting media.

DNA extraction from the transgenic plants was performed using the CTAB method. The total DNA of transformed potato as a template and untransformed as a negative control. The resistant plants obtained by neomycin phosphate transferase (*NPT II*) gene of a pair of primers (NPT II-F: GCTATGACTGGGCACAACAG) and (NPT II-R: ATACCGTAAAGCACGAGGAA) using for PCR to detections and the expected fragment size was 676 bp. The PCR reaction conditions: incubated at 94°C for 3 min, followed by 35 cycles of 94°C for 45 s, 60°C for 45 s, and 72°C for 1 min, then 10 min final extension at 72°C detecting by 1% agarose gel electrophoresis. We choose the rooting potatoes for further study and named as T1/T2 from “Gannongshu 2” transformed using the vector pCPB121-miR164, and T3/T4 from “Kexin 3” transformed using the vectors pCPB-NAC262.

### Transgenic Potato Growth and Osmotic (PEG) Stress Treatment

Transgenic potatoes were propagated on liquid MS media supplemented with 3% sucrose without kanamycin. Plantlets were fixed with filter paper and grown in 150 ml flasks under a 16/8 h light/dark cycle of white fluorescent lamp at (25 ± 2)°C. After 3 weeks, the liquid media were poured out, and 50 ml of fresh MS liquid media supplemented with 3% sucrose and 20% PEG6000 (polyethylene glycol) was added. Plantlets were cultured under the same conditions for 0, 4, 8, 16, and 32 h. The roots, leafs, and stems were collected and frozen in liquid nitrogen for preservation to extract RNA.

### qRT-PCR Analysis of the Transgenic Potato Plants

Expression of *NAC* family members was assayed using qRT-PCR with SuperReal PreMix Plus (SYBER Green) (TIANGEN). Briefly, 20 μl PCR contained about 100 ng cDNA, 10 μl 2 × SuperReal PreMix Plus, 0.6 μl each primer (NAC262-F: AGGGCTTGTGAGAGGGAATT, NAC 262-R: CGTGACTTGGCGATGAATC, efla-F: CAAGGATGACCCAGCCAAG, efla-R: TTCCTTACCTGAACGC CTGT), and 0.4 μl 50 × RO × Reference Dye. The reactions were mixed gently and incubated at 95°C for 5 min, followed by 40 cycles of 95°C for 30 s, 60°C for 34 s, and 72°C for 30 s. *ef1a* gene was used as an reference gene as control for each sample. The reaction was performed using the Mx3005p Real-Time PCR System. The relative expression level of target genes was determined using the 2^-ΔΔC_t_^ method. All samples were performed in three biological replicates with three technical replicates. The standard deviations of the data were obtained from three independent experiments. Statistical significance of individual gene expression differences upon osmotic stress was analyzed with one-way analysis of variance (ANOVA) using statistics software SPSS version 13.0.

### Physiological Assay of the Transgenic Potato Plants

The transgenic and non-transgenic potato plants were propagated by sub-culturing using single-node cuttings on MS media supplemented with 3% sucrose. Plants were grown in 150 ml flasks under (25 ± 2)°C and 2500 lx light. After 3 weeks growth, the lateral root number and length was determined. The experiments had five biological replicates. Significant differences were assayed by the Duncan’s Multiple Range Test at the 5% probability level.

## Results

### The Prediction of Stu-mi164 Target Genes

Most miRNAs are known as negative regulators of genes. miR161, miR168, miR169, miR171a, and miR319c of *Arabidopsis thaliana* are downregulated under drought stress (Sunkar and Zhu, 2004; Liu et al., 2008), and in rice and maize, miR156 is downregulated under drought stress (Wei et al., 2009; Zhou et al., 2010). The short sequence length of these miRNAs makes it relatively easy for them to combine base pairs of other sequences, potentially allowing multiple gene regulation (Ferdous et al., 2015). We reason that similar miRNAs may be present in potato with the potential to regulate the response to drought stress. The potato Stu-mi164 sequence was obtained from the miRNA registry, miRBase, and the secondary hairpin structures of Stu-mi164 is shown in Supplementary Figure S2. We used the online program psRNATarget to identify three predicted target genes *StNAC262* (XM_006364522.1), *StNAC083* (XM_00635471 0.1), and *StNAC280* (NM_001288405.1) from *Solanum tuberosum* (Supplementary Table S1). Since there are three possible targets, it is difficult to ascertain the biological role of the miRNA, and therefore, we adopted a transgenic approach to determine the role of Stu-mi164 and its three putative target genes.

### Phylogenetic Analysis of Stu-mi164 Target Gene NAC TFs

In order to know more about the evolutionary relationship of the three NAC TFs, which we predicted as the target genes of Stu-mi164, all the *StNAC* and *AtNAC* TFs were subjected to multiple sequence alignments using the CluxtalX 1.8 and MEGA 5.0 program. The multiple sequence alignment file was used to construct unrooted phylogenetic tree by the neighbor-joining method. As shown in Supplementary Figure S3, we found that the three target genes all belong to the *NAM* subfamily of potato NAC TFs. *StNAC262* and *StNAC083* are highly conserved and share a highly similar gene structure, while the *StNAC262* gene structure seemed to have experienced changes during evolution, but its coding protein still has the characteristics of NAC TFs.

### Gene Structure Analysis of *StNACs*

**FIGURE 1:**
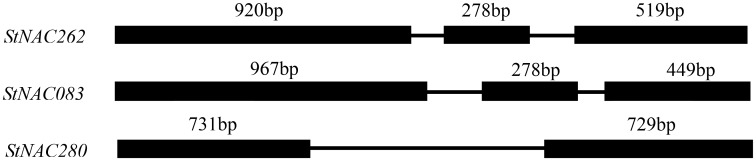
Structures of *StNACs* genes in potato. Introns and exons were represented by lines and black boxes, respectively. The numbers above the exons indicate the length (bp) of the exons.

The analysis of chromosome structure showed that *StNAC083* was located on the 6th chromosome, *StNAC262* on the 3rd chromosome, and *StNAC280* on the 7th chromosome. The result of comparison between the genomic and cDNA sequences indicated that *StNAC262* and *StNAC083* had three exons, while *StNAC280* had two exons (**Figure 1**). *StNAC262* had one 920 bp length first exon, while *StNAC083* had one 967 bp first exon. The two *StNAC* genes with three exons had a 278 bp highly conserved second exon, while *StNAC280* did not have this exon. The predicted secondary structure of the corresponding proteins suggested that the proteins derived from *StNAC262* and *StNAC083* share a high degree of structural similarity (**Table 1**). Multiple sequence alignment with the *StNAC262* protein shows high homology with *Solanum lycopersicum SlNAC100* (96%), *Nicotiana sylvestris NsNAC100-like* (84%), and *Nicotiana tomentosiformis NtNAC100-like* (82%) (Supplementary Figure S4).

**Table 1:** Basic properties and characteristics of the three *StNACs* genes in potato.

Gene	Chr	NCBI GI	Genomic locus	Cds (bp)	Exons (No)	Full length (bp)	Amino acid (aa)	pI	Mw (Da)
*StNAC262*	3	565398031	PGSC0003DMT400050262	1002	3	1714	333	8.31	38337.5
*StNAC083*	6	565376561	PGSC0003DMT400083083	1011	3	1694	336	8.13	37842.3
*StNAC280*	7	568215643	PGSC0003DMT400032280	1062	2	1523	353	8.34	39761.6

### Nucleus Localized of NAC262–EGFP Fusion Protein

**FIGURE 2:**
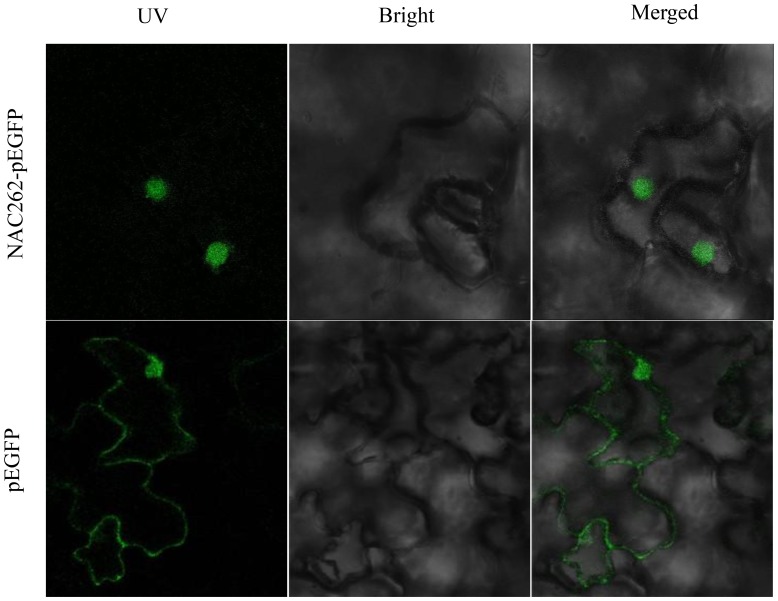
Subcellular localization of the StNAC262 protein. StNAC262 is localized in nucleus. *Agrobacteria* carrying pEGFP-NAC262 or pEGFP empty vector were infiltrated into leaves of *Nicotiana benthamiana* plants expressing and leaf samples were collected at 48 h in darkness after infiltration for observation under a confocal laser scanning microscope. Images were taken in dark field for green fluorescence (Left), white field for cell morphology (Middle) and in combination (Right), respectively.

To examine the subcellular localization of *StNAC* genes, we developed NAC-EGFP fusion proteins of StNAC262, StNAC083, and StNAC280. *Agrobacterium tumefaciens* carrying the StNAC262-EGFP fusions (or EGFP as a negative control) plasmid were infiltrated into 4-week-old *Nicotiana benthamiana* plant leaves. The result of confocal micrographs showed that the EGFP-NAC262 fusion protein was solely and clearly localized to the nucleus, and the GFP alone distributed ubiquitously throughout the cell, and no specific compartmental localization (**Figure 2**). However, the *StNAC083* and *StNAC280* gene fusions did not exhibit any recognizable localization pattern; it is possible that these two sequences were pseudogenes with no function. The data demonstrated that the StNAC262 protein was localized to nucleus of the tobacco cells.

### Expression of *StNAC262* Is Controlled by Stu-mi164 and It Is Overexpressed Under PEG Stress

**FIGURE 3:**
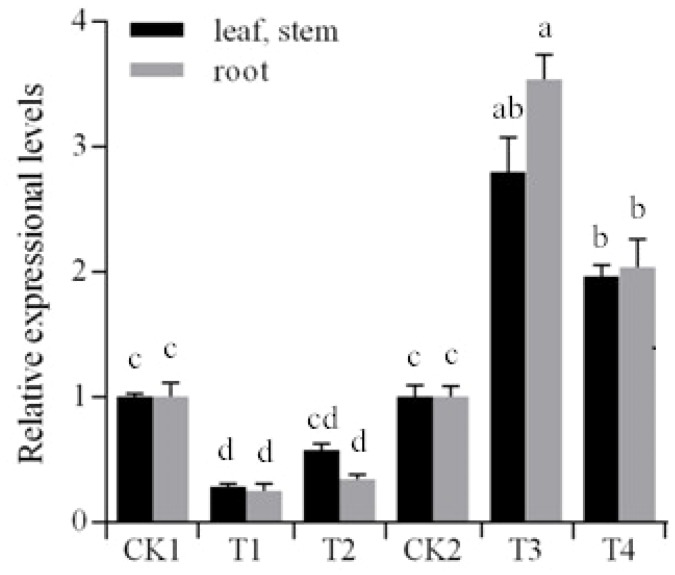
Expressional levels of *StNAC262* gene between the transgenic and non-transgenic potato plants. CK1: non-transgenic potato “Gannongshu 2” as negative control; T1–T2: the transgenic potato plant transformed with the vector pCPB121-miR164; CK2: non-transgenic potato “Kexin 3” as negative control; T3–T4: the transgenic potato plant transformed with the vector pCPB-NAC262. Data presented as a mean ± SD (three biological replicates), different letters above the columns indicate significant differences at *p* ≤ 0.05 levels with corresponding CK.

We first wanted to determine whether overexpression of Stu-mi164 would affect *StNAC262* expression. The transgenic potato plants (T1 and T2 overexpressing miR164, and T3 and T4 overexpressing *StNAC262*) and the non-transgenic potato plants were propagated *in vitro* by sub-culturing single-node cuttings on MS media containing 3% sucrose and cultured for 3 weeks. qRT-PCR analysis demonstrated that the expression of *StNAC262* gene in the transgenic potato lines T1 and T2, which overexpress miR164, was downregulated (0.58- to 0.25-fold) compared with the control, particularly in the roots of T1 (0.25-fold) (**Figure 3**). On the other hand, the expression of *StNAC262* in T3 and T4, which overexpress *StNAC26*, was upregulated (1.97- to 3.53-fold); the roots of T4 plants were upregulated 3.53-fold. This data indicated to us that indeed Stu-mi164 regulates expression of *StNAC262* (**Figure 3**).

**FIGURE 4:**
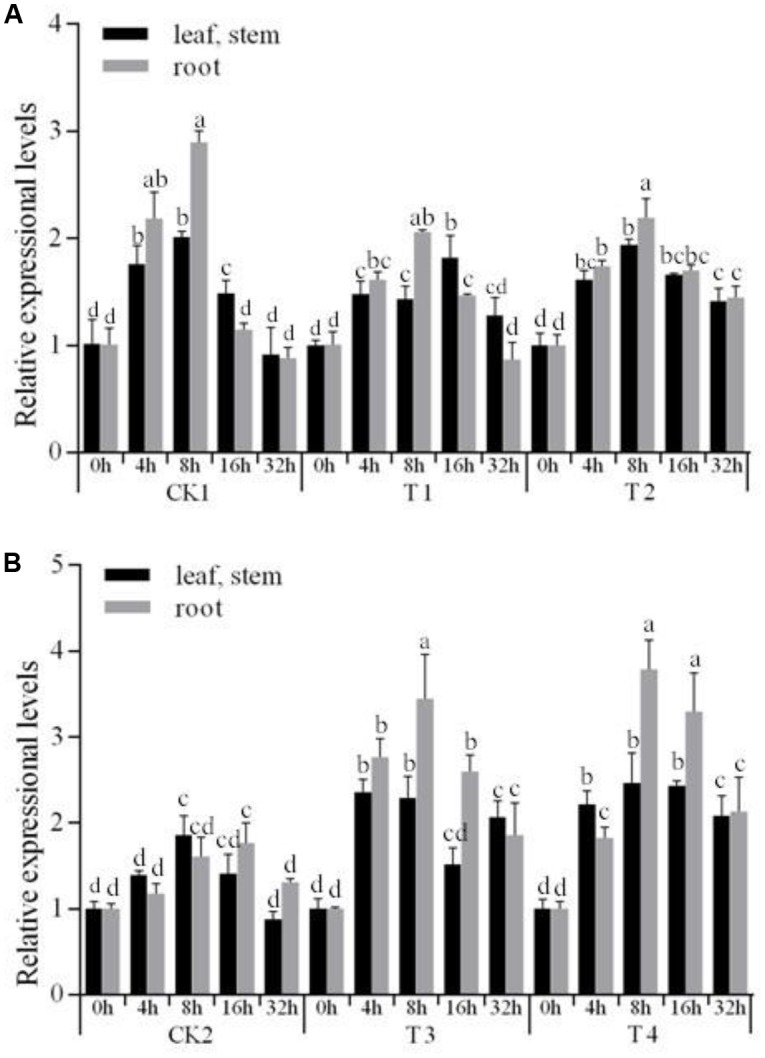
Expression levels of *StNAC262* gene under 20% PEG6000 stress treatment. Relative expressional levels of *StNAC262* in response to osmotic stress treatment for 0, 4, 8, 16, and 32 h. **(A)** qRT-PCR analysis of potato “Gannongshu 2.” CK1: non-transgenic potato “Gannongshu 2” as negative control; T1–T2: the transgenic potato plant transformed with the vector pCPB121-miR164. **(B)** qRT-PCR analysis of potato “Kexin 3.” CK2: non-transgenic potato “Kexin 3” as negative control; T3–T4: the transgenic potato plant transformed with the vector pCPB-NAC262. Data presented as a mean ± SD (three biological replicates), different letters above the columns indicate significant differences at *p* ≤ 0.05 levels with corresponding CK.

We then wanted to test whether *StNAC262* was overexpressed under PEG stress, similarly to the overexpression of the three *NAC TFs* genes in *Arabidopsis thaliana* (Tran et al., 2004). We selected five stress-treatment durations and compared their expression in the *StNAC262* transgenic and WT plants under 20% PEG6000 conditions. As shown in **Figure 4**, after 8 h under stress they reached the highest level of expression, in addition, the expression in roots was higher than leaf and stem. Furthermore, expression of *StNAC262* in transgenic miR164 plants was downregulated under PEG stress (**Figure 4A**), while the drought stress increased significantly the expression of *StNAC262* in transgenic *StNAC262* plants compared with those in WT plants (**Figure 4B**). Taken together, the data indicated that Stu-mi164 was negative regulator of gene expression in transgenic potato and that overexpression of Stu-mi164 in potato suppresses the expression of *StNAC2* and thus it conferred resistance to osmotic stress.

### Changes in Lateral Roots Associated With the Transgenic Potato Plants

**FIGURE 5:**
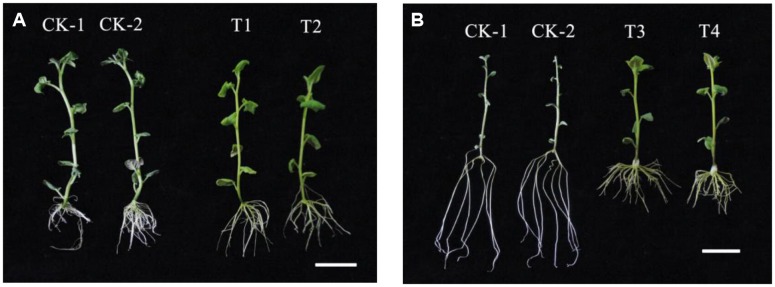
The phenotypic characteristics of the transgenic potato plants. **(A)** The transgenic potato cultivar “Gannongshu 2.” CK: non-transgenic potato as negative control; T1–T2: the transgenic potato plant. **(B)** The transgenic potato cultivar “Kexin 3.” CK: non-transgenic potato as negative control; T3–T4: the transgenic potato plant. The scale bars are of both 2 cm.

**FIGURE 6:**
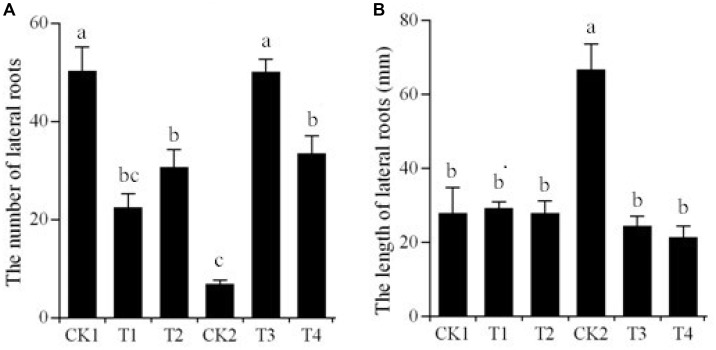
Changes of lateral root number and length of the transgenic potato plants. **(A)** The number of lateral roots. CK1: non-transgenic potato “Gannongshu 2” as negative control; T1–T2: the transgenic potato transformed with the vector pCPB121-miR164; CK2: non-transgenic potato “Kexin 3” as negative control; T3–T4: the transgenic potato plant transformed with the vector pCPB-NAC262. **(B)** The length of lateral roots. CK1: non-transgenic potato “Gannongshu 2” as negative control; T1–T2: the transgenic potato transformed with the vector pCPB121-miR164; CK2: non-transgenic potato “Kexin 3” as negative control; T3–T4: the transgenic potato plant transformed with the vector pCPB-NAC262. Values are shown as mean ± SD from five biological replicates and different letters above the columns indicate significant differences at *p* ≤ 0.05 levels with corresponding CK.

We successfully generated transgenic “Kexin 3” plants with the vector pCPB-NAC262, and transgenic “Gannongshu 2” plants with the vector pCPB121-miR164. These transgenic potato plants, which were propagated on MS media supplemented with 3% sucrose, had significant changes with respect to the lateral roots, not only in length but also in number (**Figures 5**, **6**). The overexpressions of *StNAC262* in “Kexin 3” result in a high number of lateral roots although shorter than the control, while overexpressions of miR164 in “Gannongshu 2” result in a low number of lateral roots although the same length as the control (**Figure 4**). These results are remarkable considering the fact that the potato cultivar “Gannongshu 2” naturally develops more lateral roots while “Kexin 3” naturally develops fewer lateral roots.

## Discussion

The level and activity of NAC TFs must be properly controlled for normal plant development and growth, and the mechanisms regulating NAC activity are becoming apparent (Laufs et al., 2004; Peaucelle et al., 2007; Larue et al., 2009). In the case of potato, predicted target genes *StNAC262*, *StNAC083*, and *StNAC280* and their coding proteins have the characteristics of NAC TFs. In this study, we showed that only one of the genes shows subcellular nuclear localization in potato; we have no indication that the other two genes are functional and could be pseudogenes. Knowledge of NAC TFs target genes is still limited; however, it was reported that NAC1 regulated the expression of two auxin-responsive genes *DBP* and *AIR3* (Xie et al., 2000). In the case of potato, it is likely that the genes involved in lateral root development are targets of *StNAC262*.

The same miRNA in the same plant species under osmotic stress is differentially expressed which may from the result of different spatial-temporal manner. The target genes of *Arabidopsis thaliana* miR164 family are five genes which coding *NAM/ATAF/CUC (NAC)* structure domain protein mRNA (Laufs et al., 2004). Many conserved miRNAs, such as miR156, miR159, or miR164, have proved to target stress-associated MYB and NAC family members TFs across monocots and dicots (Burcu et al., 2016). Plants excessive expressed miR164a and miR164b cotyledon and floral organ appear the phenomenon of fusion phenotypes which is similar to the *CUC1* and *CUC2* double mutant phenotypes, at the same time *CUC1* and *CUC2* transcript is decreased obviously (Ohnishi et al., 2005). *Arabidopsis thaliana* miR164 plays an important role in the process in regulating the meristem border size, differentiation of embryo and formation of floral organ (Ohnishi et al., 2005). miR164 identified from drought-stress roots of wheat can be used as candidate to develop tolerant variety to explore and exploit the drought response for future studies (Akpinar et al., 2015). The mutant of *Arabidopsis thaliana* miR164a and miR164b expressed lower miR164 and higher *NAC1* mRNA levels lead to more lateral root occurred, while induced expression in wild type plants expressed miR164 can lead to *NAC1* mRNA levels suppressed, and then reduce the occurrence of lateral root (Tang et al., 2012).

Researches (He et al., 2005; Quach et al., 2014; Chen et al., 2016) demonstrated that a number of abiotic stress upregulated NAC proteins can promote growth or alter architecture of root, when they are constitutively overexpressed in the transgenic plants, suggesting *NAC* gene roles in modulating growth and architecture of root under osmotic stress. For instance, the drought-resistant rice variety had a larger and more highly branched root system than the drought-sensitive variety (Price et al., 1997). We also found that the transgenic potato plants with *StNAC262* gene had larger root system with longer and more lateral roots than the WT plants when they were grown under osmotic stress (**Figures 5**, **6**), indicating a possible role for *StNAC262* regulating root system under osmotic stress and thereby improving tolerance to osmotic stress. It is reported that the conserved features of miRNA upon osmotic stress in *Triticeae* (Budak and Akpinar, 2011). The requirement of complementarity for target recognition, contributed to identify the putative targets of miRNAs in plants (Budak and Akpinar, 2015). It is likely that miRNA genes can change their expression under drought conditions, which in turn leads to the change in miRNAs expression and ultimately that of miRNAs’ targets (Reyes and Chua, 2007; Trindade et al., 2010). Therefore, in individual plant species, the miRNA targets need to be identified. Target validation can also provide functional evidences for the conserved or specific miRNAs in plant.

The potato cultivar “Gannongshu 2,” which naturally produces more lateral roots, and “Kexin 3,” which naturally produces fewer lateral roots, were used in the experiments reported here. Overexpression of *StNAC262* in the Kexin 3 variety produced an increased number of lateral roots thus completely changing the natural tendency of this variety to now produce less lateral roots. Likewise, overexpression of Stu-mi164 in the “Gannongshu 2” variety developed a reduced number of lateral roots thus completely changing the natural tendency of this variety to now produce less lateral roots. So, it appears that a mechanism to deal with osmotic stress in potato is through the production of extra lateral roots, which is a process controlled by *StNAC262* and its modulator Stu-mi164. Plants having a more developed root system possess a greater ability to absorb water, so the osmotic stress plants can absorb more water from the soil to reduce the damage caused by a lack of water. Therefore, it is possible to improve the resistant ability of plants to osmotic stress by changing the lateral root number.

## Author Contributions

HS and NZ conceived and designed the experiments. LZ, LY, JY, XZ, and XT performed the laboratory experiments. LZ, LY, NZ, and AC-U performed the data analysis and interpretation. LZ, LY, AC-U, and HS wrote the paper.

## Conflict of Interest Statement

The authors declare that the research was conducted in the absence of any commercial or financial relationships that could be construed as a potential conflict of interest.

## Footnotes

1 http://microrna.sanger.ac.uk/

2 http://plantgrn.noble.org/psRNATarget/

3 http://wmd3.weigelworld.org/cgi-bin/webapp.cgi

4 http://planttfdb.cbi.pku.edu.cn/

5 http://solanaceae.plantbiology.msu.edu/pgsc_download.shtml

6 http://www.ncbi.nlm.nih.gov
