# Supplementary material for: Lateral Root Development in Potato Is Mediated by Stu-mi164 Regulation of NAC Transcription Factor
Source: Front Plant Sci. 2018 Mar 29;9:383. doi: 10.3389/fpls.2018.00383 (PMC5884874; doi:10.3389/fpls.2018.00383)
Supplement: Supplementary file 1 [file Table_1.DOCX]

**Supplementary Table S1.** Predicted target genes of Stu-miR164 and alignment

| **miRNA Acc** | **Target Acc** | **Alignment** |
| --- | --- | --- |
| TGGAGAAGCAGGGCACATGCT | PGSC0003DMT400032280 | miRNA 21 UCGUACACGGGACGAAGAGGU 1  : : : : : : : : : : : : : : : : : : :  Target 1037 AGCACGUGCCCUGUUUCUCCA 1057 |
|  | PGSC0003DMT400050262 | miRNA 20 CGUACACGGGACGAAGAGGU 1  : : : : : : : : : : : : : : : : :  Target 935 UCACGUGCACUGCUUCUCCA 954 |
|  | PGSC0003DMT400083083 | miRNA 20 CGUACACGGGACGAAGAGGU 1  : : : : : : : : : : : : : : : : :  Target 880 CCACGUGCACUGCUUCUCCA 899 |
