# Supplementary material for: Lateral Root Development in Potato Is Mediated by Stu-mi164 Regulation of NAC Transcription Factor
Source: Front Plant Sci. 2018 Mar 29;9:383. doi: 10.3389/fpls.2018.00383 (PMC5884874; doi:10.3389/fpls.2018.00383)
Supplement: Supplementary file 2 [file Image_1.PDF]

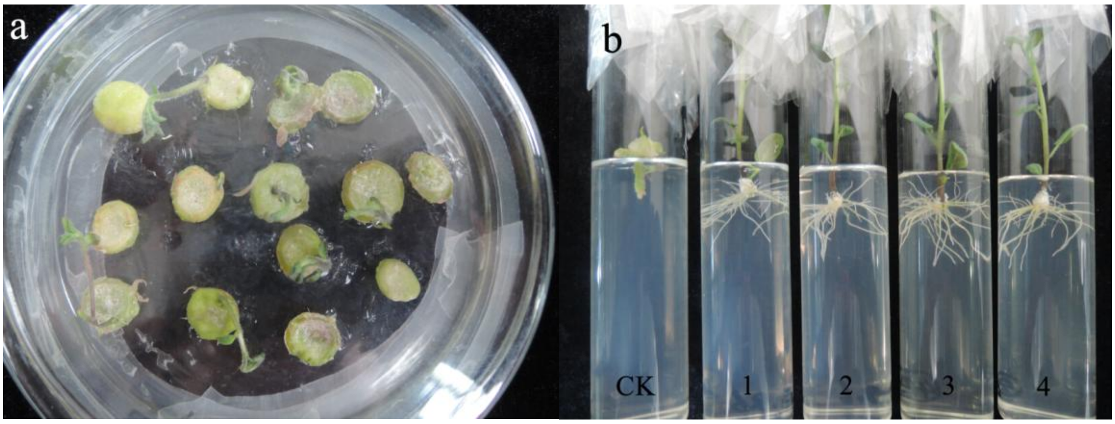

Supplementary Figure S1. Transgenic potato plants development and screening on the rooting medium containing kanamycin. **a**. Shoots formation directly from transgenic microtuber discs of the potato ‘‘kexin 3’’ after three weeks of culture in the selective medium and incubated under a photoperiod with 16 h light/8 h dark cycles at 25 ^{o}C. **b**. The roots were formed in about 20 days when green shoots were transferred to the selective rooting medium. CK: untransformed potato as negative control; 1,2: the pCPB121-miR164 transgenic potato line; 3,4: the pCPB-NAC262 transgenic potato plant lines.

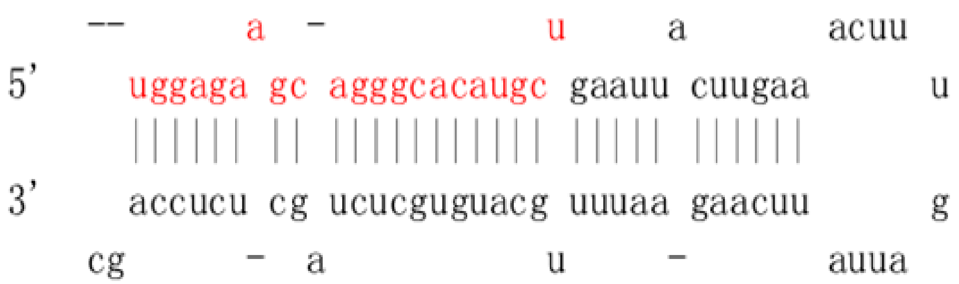

Supplementary Figure S2. Secondary hairpin structures of Stu-miR164 in potato. Mature miRNA sequences are in red.

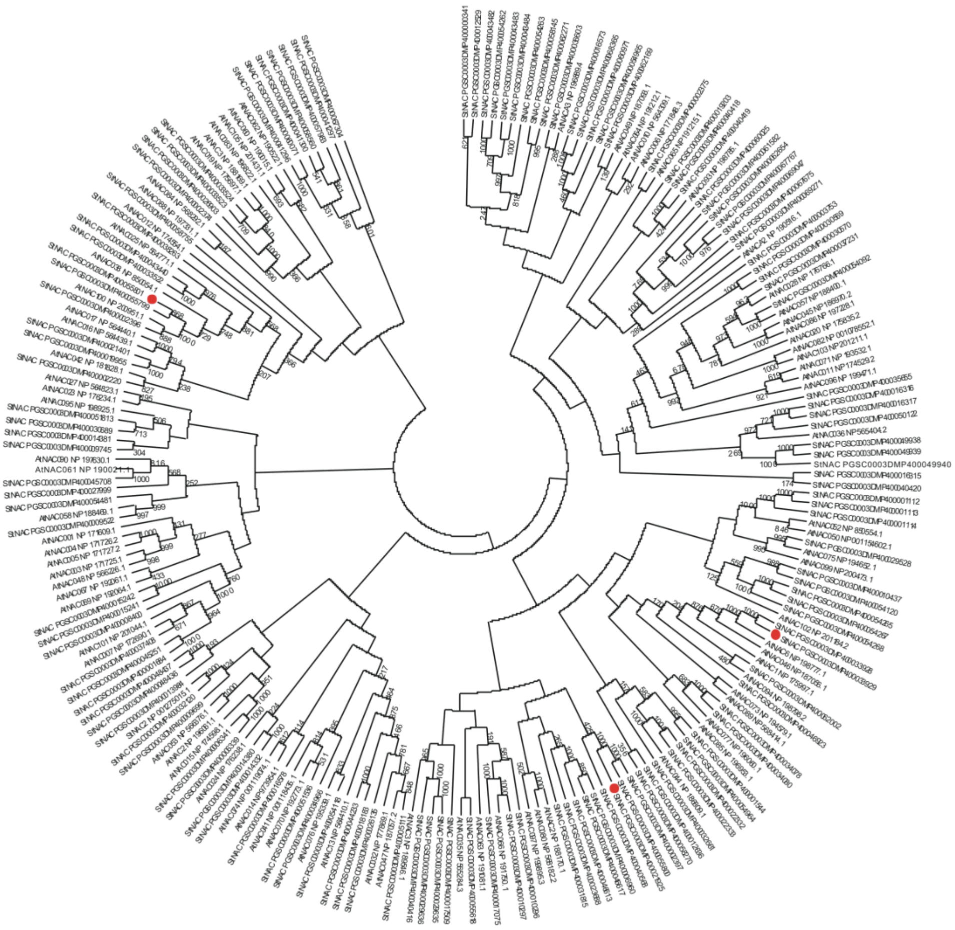

Supplementary Figure S3. Phylogenetic tree constructed with CluxtalX1.8, using the *NAC* full length amino acid sequence from *Arabidopsis thaliana* and *Solanum tuberosum*. The location of the target genes was marked with red dot.

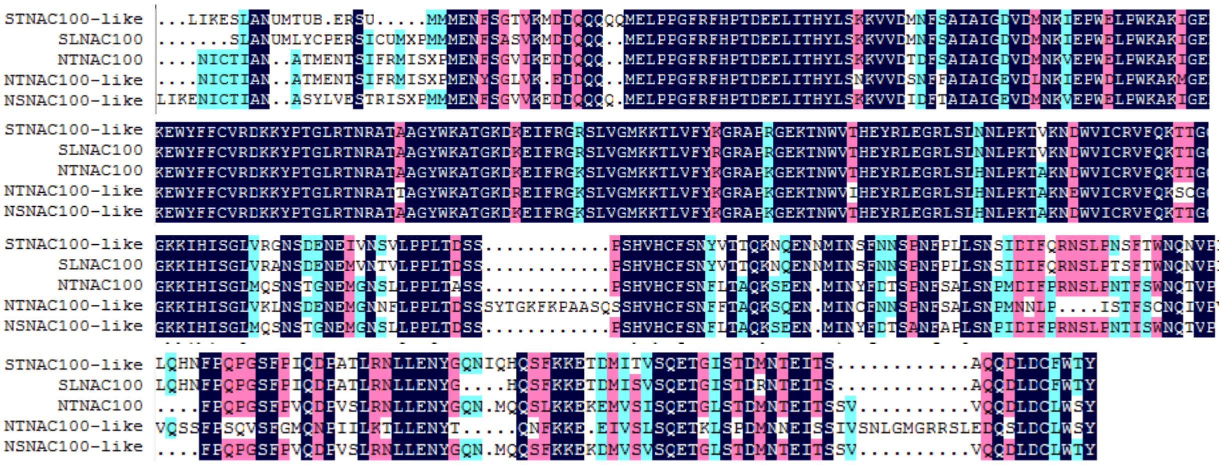

Supplementary Figure S4. Multiple alignment of the StNAC protein sequence from various plants. Identical amino acid residues are covered by black, similar residues are indicated by colour, and the gaps in the sequences are indicated by dashes. STNAC100-like: *NAC262* in *Solanum tuberosum*, SLNAC100: *NAC100 in Solanum lycopersicum*, NTNAC100-like: *NAC100-like in Nicotiana tomentosiformis*, NTNAC100: *NAC100* in *Nicotiana tomentosiformis*, NSNAC100-like: *NAC100-like* in *Nicotiana sylvestris*.
